# Increasing numbers of harbour seals and grey seals in the Solent

**DOI:** 10.1002/ece3.8167

**Published:** 2021-11-18

**Authors:** Robyne Castles, Fiona Woods, Peter Hughes, John Arnott, Louise MacCallum, Sarah Marley

**Affiliations:** ^1^ Institute of Marine Sciences University of Portsmouth Portsmouth UK; ^2^ Chichester Harbour Conservancy Itchenor UK; ^3^ Langstone Harbour Board Hayling Island UK; ^4^ Scotland's Rural College (SRUC), Craibstone Estate Aberdeen Scotland

**Keywords:** grey seals, harbour seals, photographic identification, population dynamics

## Abstract

Harbour seals (*Phoca vitulina*) and grey seals (*Halichoerus grypus*) both occur within the UK, but display regional contrasting population trends. While grey seals are typically increasing in number, harbour seals have shown varying trends in recent decades following repeated pandemics. There is a need for monitoring of regional and local populations to understand overall trends. This study utilized a 20‐year dataset of seal counts from two neighboring harbours in the Solent region of south England. Generalized additive models showed a significant increase in the numbers of harbour (mean 5.3–30.5) and grey (mean 0–12.0) seals utilizing Chichester Harbour. Conversely, in Langstone Harbour there has been a slight decrease in the number of harbour seals (mean 5.3–4.0). Accompanying photographic data from 2016 to 18 supports the increase in seal numbers within Chichester Harbour, with a total of 68 harbour and 8 grey seals identified. These data also show evidence of site fidelity of harbour seals in this area, with almost a quarter of animals resighted within the past three years. Overall, this long‐term study indicates an increasing number of both harbour and grey seals within the Solent. However, more research is required to identify the drivers of this trend.

## Graphical abstract

A 20‐year dataset from the Solent (south England) showed an increasing number of harbour and grey seals over time. This area may be particularly important for harbour seals, as they use it for pupping in the summer months.

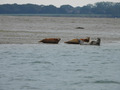

## 1 INTRODUCTION

**FIGURE 1:**
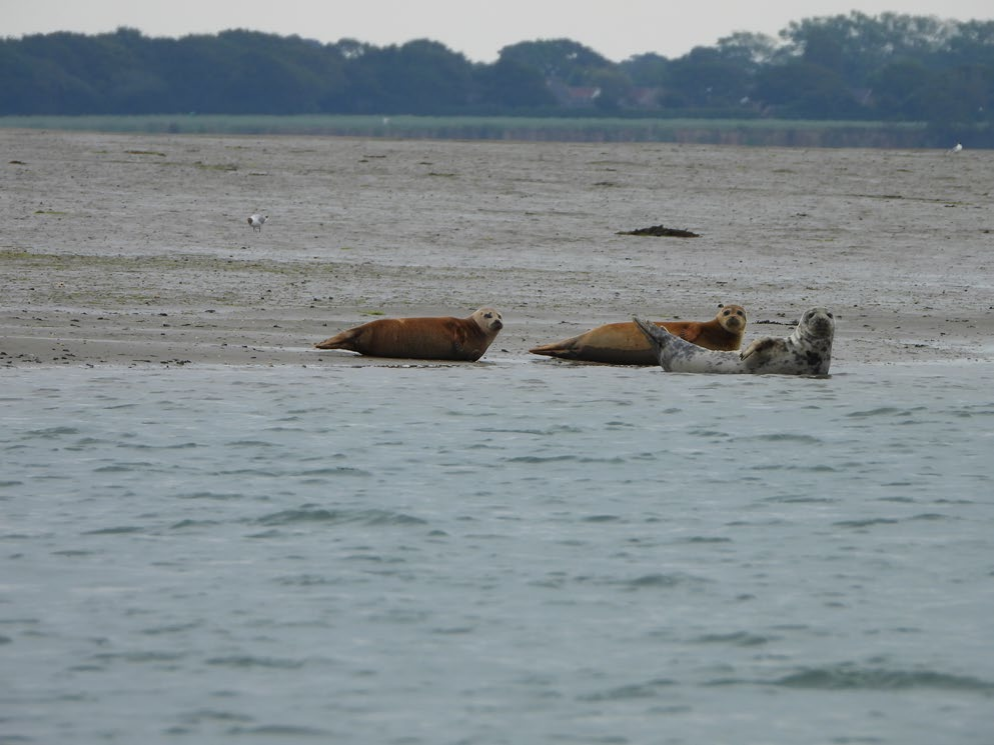
Two harbour seals (*Phoca vitulina*) and a grey seal (*Halichoerus grypus*) hauled out in Langstone Harbour, south England (credit: Sarah Marley)

Harbour seals (*Phoca vitulina*) and grey seals (*Halichoerus grypus*) are widely distributed in the temperate North Atlantic and currently listed as species of “Least Concern” (Figure 1; IUCN, 2019). However, within the UK there are contrasting population trends for these species.

Harbour seals were severely impacted by phocine distemper virus (PDV) in 1988 and 2002, which resulted in up to 50% reductions in some UK subpopulations (SCOS, 2020). Recent results indicate that the overall UK harbour seal population has now recovered to levels similar to those of the late 1990s (estimated 44,100 animals; 95% CI 36,100–58,800); however, there have been significant regional differences in trends, with various subpopulations decreasing, stable, or increasing (SCOS, 2020; Thompson et al., 2019). In comparison, the UK grey seal population size is estimated to be 150,000 animals, with regular surveys of grey seal pup production showing an increasing trend since surveys began in the 1960s (Russell et al., 2019; SCOS, 2020). However, there has been a notable reduction in the growth rates of some grey seal subpopulations in recent years (Thomas et al., 2019).

Therefore, it is not sufficient to merely examine the total size of a population; long‐term monitoring of regional and local subpopulations is required to fully understand overall population trends and rates of change. This is particularly true in the case of new or small subpopulations. In the UK, such monitoring is important for meeting various conservation legislation and management requirements. However, in a more applied sense, monitoring at various spatial scales is also important for the mitigation of mounting anthropogenic threats in the form of fisheries by‐catch, vessel traffic, marine pollution, underwater noise, marine renewable energy, tourism, and climate change (Alexander et al., 2016; Boitani et al., 2011; Coomber et al., 2016; Erbe et al., 2019; Hines et al., 2020; Papageorgiou, 2016; Partelow et al., 2015; Payne et al., 2016). Understanding seal abundance at a national, regional, and local level is necessary to support effective management and conservation at a range of scales.

Within the UK, the harbour seal population is subdivided into 14 seal management units (SMUs), which were originally defined on the basis of the spatial distribution of haul‐out sites and knowledge of harbour seal ecology but also appear to be in agreement with results from genetic population structure studies (Olsen et al., 2014; Thompson et al., 2019). A detailed analysis of long‐term trends for several SMUs is presented in Thompson et al. (2019) and summarized in SCOS (2020), demonstrating contrasting dynamics between subpopulations on the English east coast (increasing year on year), east coast of Scotland and in the Northern Isles (declines of varying intensity), and the western UK (stable or increasing). However, there is no clear explanation for these differing dynamics (Thompson et al., 2019). Unfortunately, SMUs #10–13 in south England, southwest England, Wales, and northwest England could not be included in these analyses due to limited information, as previous reports suggest harbour seals to be effectively absent from this stretch of coastline (SCOS, 2017). Thus, these SMUs have not been regularly surveyed for harbour seals (Thompson et al., 2019), but this risks overlooking important changes at the local and regional level.

Such a change appears to have occurred in SMU #10 (south England), where harbour and grey seals have reportedly formed new haul‐outs in the Solent region. In 1994, three harbour seals were recorded using Chichester Harbour, followed by an increasing number of sightings over time. A dedicated local research project in 2009 collated existing information on seals in the area and deployed satellite tags on five harbour seals to investigate residency and movement patterns (Chesworth et al., 2010). This identified two significant haul‐out sites, one in Chichester Harbour and one in neighboring Langstone Harbour, with approximately 24 harbour seals reported across both sites. The tagged harbour seals appeared to be resident in the area, remaining almost entirely within the eastern Solent and repeatedly visiting both the Chichester and Langstone Harbour haul‐out sites. In July 2008, the first official sighting of a grey seal was also reported at the haul‐out site in Chichester Harbour (Chesworth et al., 2010). The report did not hypothesize the drivers behind this increase in seal abundance (i.e., reproduction vs. immigration), although anecdotal evidence exists of harbour and grey seals being present in the area for several decades prior and their numbers may also have been supplemented by the introduction of rehabilitated animals.

Regardless of where these seals have come from, their relatively low numbers and proximity to human activities raise considerable concern. The Solent has many anthropogenic uses, including recreational activities, commercial shipping from Portsmouth and Southampton harbours, fisheries and marine aggregate extraction, and utilization by the Royal Navy (Conway, 2007). In addition, the surrounding land area is highly urbanized. However, there is a paucity of literature regarding the current size of the Solent seal haul‐out, whether pups are being produced at this site, or the potential impact of anthropogenic activities. Obtaining updated estimates of animal numbers and potential trends is a fundamental first step to managing seals within this SMU.

Our study aimed to assess the seal population trends in Chichester and Langstone Harbours using a 20‐year dataset and three years of photographic identification (photo‐ID) data. Building upon the 1999 to 2008 sighting dataset in Chesworth et al. (2010), we collated additional survey data collected by local harbour authorities from 2009 to 2019. We then fitted generalized additive models (GAMs) to model seasonal and long‐term trends in these count data. From 2015 onward, surveys were expanded to include photo‐ID, which we reviewed to investigate the number of individuals present in this area and the possibility of site fidelity. Outcomes from this research will provide managers with a much‐needed update on seal abundance and site use within the Solent, and will also be relevant for guiding future monitoring of the south England SMU.

## 2 MATERIALS AND METHODS

### 2.1 Study area

**FIGURE 2:**
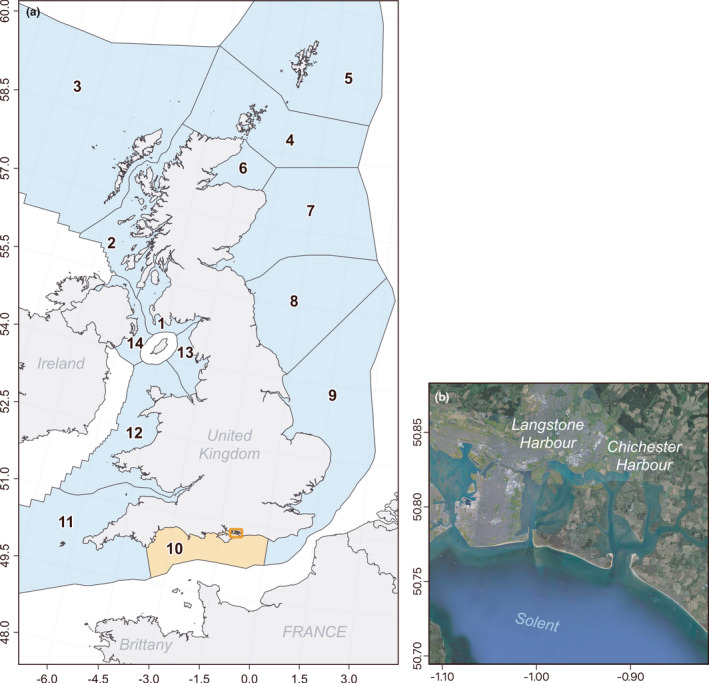
Map of (a) numbered seal management units (SMUs) in the UK and (b) the two Solent study sites located in SMU #10

The Solent is a sheltered channel system, separated from the English Channel by the Isle of Wight and stretching along the coastline of Hampshire and West Sussex (Figure 2). It includes numerous estuaries and natural harbours, the largest of which are Portsmouth, Langstone, and Chichester Harbours, which form a series of connected basins comprised of extensive intertidal mud and sandbanks. Previous studies have confirmed that harbour seals utilize haul‐outs in both Chichester and Langstone Harbours, moving between these sites via both the sea and a network of tidal channels (Chesworth et al., 2010). Grey seals have previously only been reported in a single sighting located within Chichester Harbour (Chesworth et al., 2010).

### 2.2 Data collection

Visual surveys were undertaken in Chichester Harbour from 1999 to 2012 and 2015 to 2019, with surveys in Langstone Harbour from 2009 to 2017 and 2019 (Appendix 1). Surveys approximately overlapped with low tide, when the tidal mudflats were at their maximum availability for seal haul‐outs. However, in some cases surveys were limited by poor weather.

Pre‐2015, data were collected on a monthly basis using a combination of boat‐ and land‐based surveys and did not include estimates of seal age. Post‐2015, field protocols at both sites were standardized to ensure the same methodology was consistently applied. This included the following: moving surveys to an entirely boat‐based platform; conducting surveys concurrently at both harbours to remove the chance of individuals being counted twice; undertaking surveys at least once a month between May and September to capture the harbour seal pupping season (with additional months surveyed on an opportunistic basis); and recording the approximate age of individuals (adult, subadult/juvenile, or pup) where possible. It can be challenging to assign an age cohort from observation alone (except for newborn pups), especially for harbour seals; despite females having lower overall lengths at maturity, they display higher early growth rates than males (Hall et al., 2019). Hence, subadults and juveniles were combined into one category. Regardlessly, age/sex data were not used in the present analyses beyond observation of pup numbers. Note that it was not possible to collect count data in Langstone Harbour during 2018.

Additionally, boat‐based photo‐ID using a digital SLR camera and appropriate zoom lens was undertaken in Chichester Harbour from 2016 to 2018. Photograph processing followed the standard protocols (Cordes & Thompson, 2015; Cunningham et al., 2009; Hastings et al., 2008; Mackey et al., 2007; Yochem et al., 1990). In brief, photographs were sorted by survey and allocated ratings based on their quality (i.e., clear focus, good lighting, and head visible) for identifying pelage markings: Grade 1 (good quality), Grade 2 (sufficient), or Grade 3 (poor). Only Grade 1 and 2 photographs were retained. Four independent reviewers manually compared images to identify individual seals based on unique pelage markings. “New” individuals were allocated a sequential identification number and added to the catalogue. When individuals were resighted, the date was noted in a separate spreadsheet.

### 2.3 Data analysis

Seal count data were analyzed in R (R Core Team, 2019) for each species and site using generalized additive models (GAMs, *mgcv* package; Wood, 2011) to allow smooth functions to be fitted to temporal covariates (Month and Year) based on results from exploratory analyses. Cyclic cubic splines were used for month to ensure there was no discontinuity between January and December. To account for overdispersion, GAMs were fitted with a Tweedie distribution (Miller et al., 2013). Restricted maximum likelihood (REML) was used to minimize overfitting (Wood, 2011). The best model was selected using Akaike's information criterion for small sample sizes (AICc; *MuMIn* package; Barton, 2020). Model assumptions were checked by producing standard residual diagnostic plots, and an acf function was used to check for temporal autocorrelation (Zuur & Ieno, 2016). See “Data Availability” section for a link to code used in these analyses.

## 3 RESULTS

Overall, 270 surveys (182 Chichester and 88 Langstone) were conducted across the 20‐year study period (Appendix 1).

### 3.1 Seal counts

**FIGURE 3:**
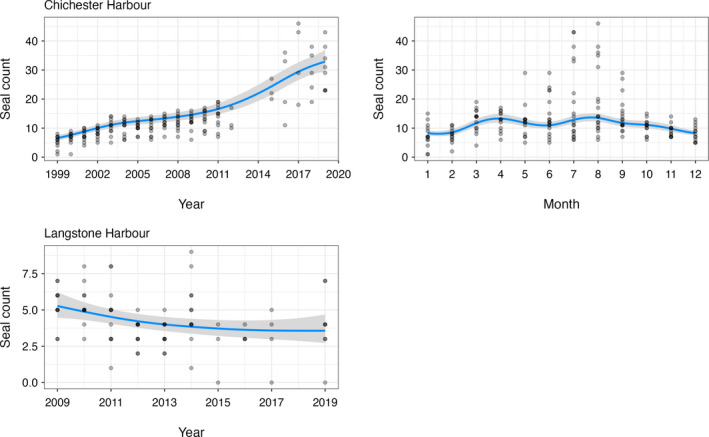
Harbour seal counts in relation to significant explanatory variables

**FIGURE 4:**
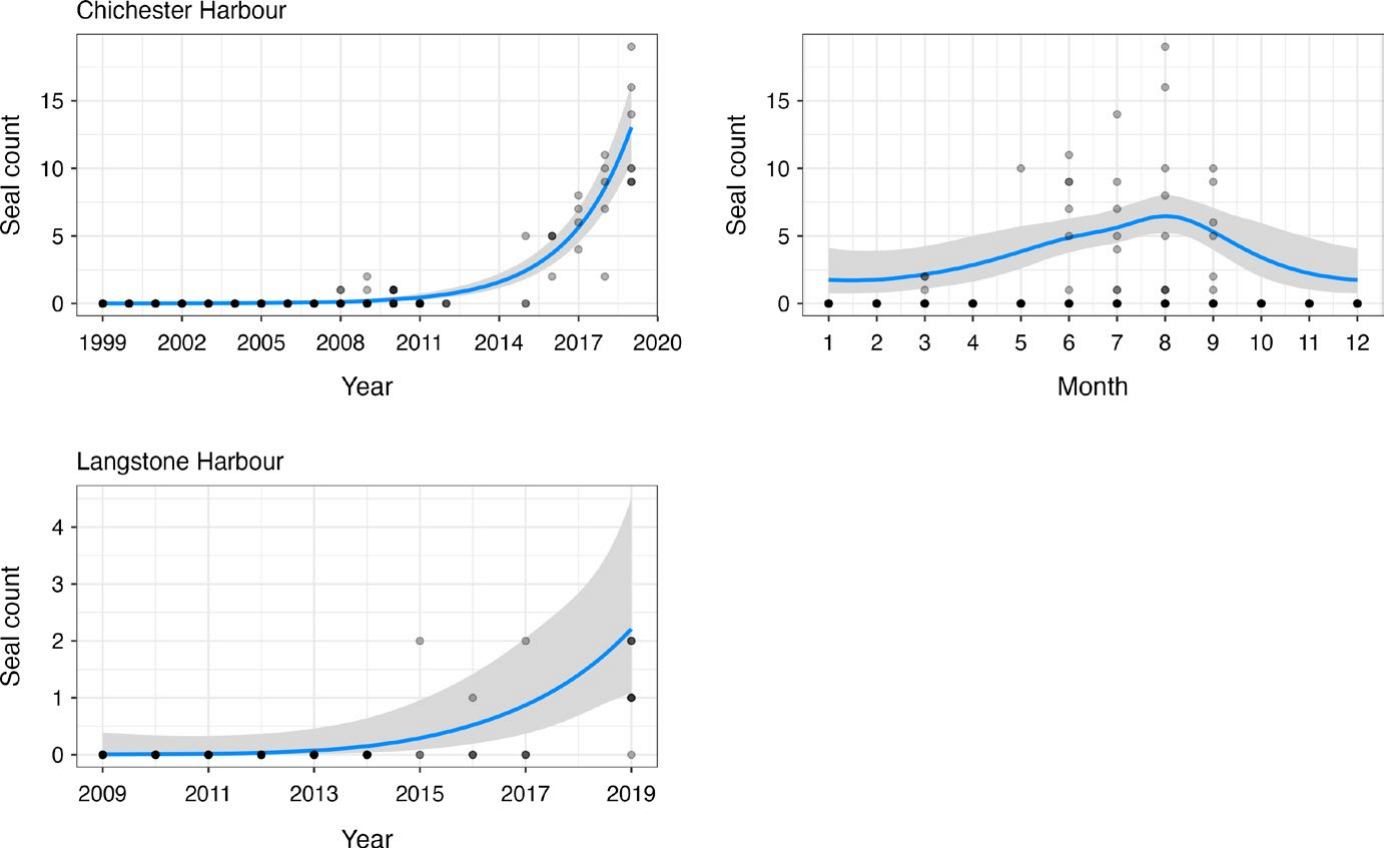
Grey seal counts in relation to significant explanatory variables

In Chichester Harbour, there has been a significant increase in the number of harbour and grey seals between 1999 and 2019 (*p* < .001; Table 1). During this time, the mean number of harbour seals has increased from 5.3 (±2.1 *SD*) to 30.5 (±7.5 *SD*) (Figure 3). Similarly, the mean number of grey seals has increased from 0 to 12.0 (±3.9 *SD*) (Figure 4). The most recent Chichester Harbour counts indicate a maximum of 43 harbour seals and 19 grey seals observed in 2019. There were also significant monthly trends in seal counts at this site for both species (both *p* < .01; Table 1). Peak numbers occurred in August for both harbour (mean 19.0 ± 12.0 *SD*) and grey (mean 3.2 ± 5.8 *SD*) seals, with harbour seals also showing a smaller peak in March (mean 12.7 ± 0.7 *SD*).

**TABLE 1:** Summary of the best‐fitting model of seal counts in relation to time. Separate GAMs were fitted according to species and site

Smooth term	EDF	*F*	*p*‐Value	Sig
Harbour seals (Chichester)				
Month	6.898	8.835	1.08e15	***
Year	4.700	103.584	<2e−16	***
Harbour seals (Langstone)				
Year	1.768	3.984	.0197	*
Grey seals (Chichester)				
Month	2.842	1.164	.00389	*
Year	1.000	171.630	<2e−16	***
Grey seals (Langstone)				
Month	2.136	0.531	.06189	
Year	1.449	8.454	.00685	**

Note

Significance levels: ≤0.05*; ≤0.01**; and ≤0.001***.

In Langstone Harbour, there has been a small but significant change in the number of harbour and grey seals between 2009 and 2019. During this time, the mean number of harbour seals has decreased from 5.3 (±1.3 *SD*) to 4.0 (±2.3 *SD*) (*p* = .0197; Table 1; Figure 3), whereas the mean number of grey seals has slightly increased from 0 to 2 (±0.7 *SD*) (*p* =.0069; Table 1; Figure 4). The most recent Langstone Harbour counts indicate a maximum of 7 harbour seals and 2 grey seals observed in 2019. There were no significant monthly trends in seal counts at this site for either species.

From 2015 to 2019 (excluding 2018), surveys were conducted at both harbours simultaneously and animal age class was also recorded. In 2015, the combined peak number of adult and subadult animals was 22 harbour and 5 grey seals; in 2016, 29 and 5 seals; in 2017, 35 and 9 seals; and in 2019, 39 and 20 seals. In addition, in these years, 8, 10, 11, and 8 harbour seal pups were recorded, respectively. Pups were only observed in Chichester Harbour. For all age classes combined, there was a maximum concurrent count of 47 harbour seals and 20 grey seals across both harbours.

### 3.2 Photo‐ID

Over three years of photo‐ID data collection in Chichester Harbour, a total of 68 harbour and eight grey seals were identified (Appendix 2; see “Data Availabilty” section for a link to the full photo‐ID catalogue). Note that it was not always possible to photograph every seal present and not all photographs were of high enough quality for individual identification. Therefore, these data likely present an underestimate of the true number of individual seals present.

For harbour seals, 29 new individuals were discovered in 2016, 10 in 2017, and 28 in 2018 (Table 2). The fact that high numbers of “new” animals were still being identified also supports the suggestion that these data are an underestimate of the number of seals using this harbour. However, there is still some evidence of site fidelity; of the 68 individuals, 16 (23.9%) were resighted within the study period (Table 3). The shortest resighting period was one month, while the longest resighting period was three years. The highest number of resightings occurred in September 2019 (i.e., the final survey), which may be due to the photo‐ID catalogue now being of a sufficient size to facilitate resightings. There were no apparent seasonal patterns in resightings, which likely reflects the short study duration; similarly, insufficient sex/age data were available to investigate potential patterns according to these factors.

**TABLE 2:** Summary of new and cumulative individual seals discovered over time from photo‐ID surveys

Survey date	Harbour seals		Grey seals	
	New	Cumulative	New	Cumulative
13‐Jun‐16	2	2	0	0
27‐Jul‐16	7	9	1	1
24‐Aug‐16	10	19	0	1
27‐Sep‐16	8	27	0	1
12‐Oct‐16	2	29	0	1
20‐Jun‐17	2	31	1	2
17‐Jul‐17	3	34	0	2
29‐Aug‐17	3	37	0	2
28‐Sep‐17	2	39	1	3
26‐Mar‐18	8	47	0	3
20‐Jun‐18	3	50	2	5
9‐Jul‐18	4	54	0	5
20‐Jul‐18	3	57	1	6
21‐Aug‐18	4	61	0	6
3‐Sep‐18	7	68	2	8

**TABLE 3:** Summary of individual harbour and grey seals resighted within Chichester Harbour in 2016–2018

ID	2016					2017				2018						Total
	13/06	27/07	24/08	27/09	12/10	20/06	17/07	29/08	28/09	26/03	20/06	09/07	20/07	21/08	03/09	
Harbour seals																
SOL‐4		X	X													2
SOL‐5		X			X						X					3
SOL‐6		X										X			X	3
SOL‐7		X										X				2
SOL‐10			X					X							X	3
SOL‐11			X		X				X						X	4
SOL‐16			X								X				X	3
SOL‐18			X						X							2
SOL‐23				X											X	2
SOL‐27				X					X							2
SOL‐28					X				X							2
SOL‐37								X							X	2
SOL‐38									X	X					X	3
SOL‐47										X					X	2
SOL‐54												X	X			2
SOL‐57													X		X	2
Grey seals																
GR‐4											X			X		2

For grey seals, one new individual was discovered in 2016, two in 2017, and five in 2018 (Table 2). One animal (an adult female) was resighted two months apart in 2018 (Table 3).

## 4 DISCUSSION

Our study revealed an overall increase in the number of harbour and grey seals within the Solent across the 20‐year study period. Both species were consistently more abundant in Chichester Harbour than Langstone Harbour, with the former area also experiencing some seasonality in peak counts and acting as a pupping site for harbour seals. We recorded preliminary evidence of site fidelity, with approximately 25% of the 68 individually identified harbour seals resighted within Chichester Harbour. However, of the eight grey seals identified, only one individual has been resighted so far.

### 4.1 Increasing seal numbers in the Solent

The first two harbour seals were reported in Chichester Harbour in 1994, and subsequent surveys estimated a total of 23–25 animals using Chichester and Langstone Harbours in 2009. The first grey seal was reported at this site in 2008 and by the following year counts had increased to two animals. Our findings show that Chichester Harbour alone is now utilized by up to 43 harbour and 19 grey seals at any one time; however, when counts from Langstone Harbour are included, this increases to 47 harbour and 20 grey seals.

However, it is worth noting that count data alone will not capture the true number of seals present. Both species undertake short foraging trips (mean 31 hr for harbour seals, Cunningham et al., 2009; mean 2.33 days for grey seals, McConnell et al., 1999), resulting in varying temporal use of haul‐out sites. To account for this, a scaling factor of 0.72 (95% CI: 0.54–0.88) is typically applied to capture the estimated proportion hauled out versus in‐water (SCOS, 2020). Applying the same scaling in the present study produces an estimate of 60 (95% CI: 49–80) harbour seals, which closely matches the results of the photo‐ID data (68 individuals). Note that there is currently no equivalent scaling factor for grey seals, so the proportion “missed” in the present surveys is unknown.

The increase in harbour seals is partly due to reproductive activities within Chichester Harbour. Several harbour seal pups were recorded in Chichester each year between 2015 and 2019, although pupping also likely occurred prior to this date, but age data were not collected in earlier surveys. In many cases, pups were only a few hours old, highlighting the importance of this site as a pupping area. However, given that photo‐ID is still regularly identifying new adult animals, immigration from other haul‐out sites is likely another contributing factor. Similarly, as grey seals are not known to pup at any location in the Solent, links with other haul‐out sites presumably account for their increasing numbers in this region.

There are no other documented harbour seal haul‐outs in the south England SMU. The closest significant UK haul‐out is in the Greater Thames Estuary, located ca. 280 km away as the seal swims in the southeast England SMU. Seal numbers have been increasing in the Thames over recent years, with the most recent surveys estimating that approximately 900 harbour and 3,200 grey seals reside throughout the estuary (Cox et al., 2020). Haul‐out sites in France represent a considerably shorter travel distance (approximately 160 km), and regular surveys along the French coast of the English Channel also show significant increases in harbour and grey seal numbers (Vincent et al., 2017). However, French telemetry studies indicate that harbour seals remained highly coastal (within 20 km from shore), close to their haul‐out site (within 100 km of their capture site), and did not visit other colonies (Vincent et al., 2017). This aligns with findings from other tracking studies, including one in Chichester Harbour, which indicate tight concentrations of harbour seals around the coastline adjacent to their haul‐out sites (Carter et al., 2020; Chesworth et al., 2010; Cunningham et al., 2009). In comparison, grey seals typically move much greater distances and regularly utilize offshore foraging areas (Carter et al., 2020; McConnell et al., 1999). Telemetry studies in France show grey seals moving up to 1,200 km from their capture site, including frequent trips across the English Channel (Vincent et al., 2017). Indeed, one of the male grey seals observed in Langstone Harbour during the present study carried man‐made markings that identified him as an individual previously rehabilitated in France. The seal was released on 3 April 2015 in Brittany and was observed in Langstone Harbour (approximately 250 km away) on 3 May 2015. Thus, such channel crossings combined with increasing subpopulations in France may account for the increasing presence of grey seals within the Solent. However, links between the Solent and other harbour seal haul‐outs around the English Channel are yet to be confirmed. Further studies utilizing telemetry and photo‐ID would also be beneficial for understanding links between the Solent seals and other sites.

### 4.2 Spatiotemporal patterns within the Solent

Within the Solent, we observed discrepancies in site use between Langstone and Chichester Harbours. The drivers behind these are unclear. Both harbours are linked to the north by a series of tidal creeks and are accessible via the open sea to the south, but potentially differ in terms of habitat quality within their confines. Chichester Harbour is an area of outstanding natural beauty (AONB), with excellent water quality ratings and primarily recreational vessel traffic. By contrast, Langstone Harbour is highly urbanized due to its proximity to Portsmouth City, experiences both recreational and commercial vessel traffic due to its two marine aggregate wharves, and also has a sewage treatment plant located at the northern end of the harbour. These characteristics may also account for why harbour seal pupping only occurs in the former site. Yet, both harbours still support a range of habitats, maintain high levels of biodiversity with regard to wading and seabirds, fish, and marine invertebrates, and are designated as bass nursery areas (CHC, 2019; LHB, 2019). A previous telemetry study recorded harbour seals regularly foraging in Langstone and Chichester Harbour, although dive locations did switch over time, suggesting that foraging patterns were related to prey availability (Chesworth et al., 2010). However, it is worth noting that Langstone Harbour has a small number of seals overall, which may accentuate small fluctuations. Further monitoring will be beneficial to establishing whether seal numbers are indeed changing at this site. It would also be interesting to explore site use within and between the harbours with regard to age/sex factors, or even individual preferences.

The present study identified significant monthly trends in seal counts, with both harbour and grey seal numbers peaking in August within Chichester Harbour. This coincides with the annual harbour seal molt, when animals spend longer periods of time ashore for thermoregulation (Paterson et al., 2012). The higher number of grey seals at this time may represent animals traveling through the area on route to breeding sites ahead of the reproductive season.

It is worth noting that the majority of the study period did not experience coordinated counts for both harbours. As earlier counts were not conducted at the same time, it is possible that individual seals could move between the harbours and be double‐counted. Coordinated counts have been undertaken from 2015 along with standardized data collection methods, providing the basis for a robust long‐term dataset. As well as monthly counts, it would also be beneficial to explore additional data collection options. Here, monthly trends in seal counts were observed within Chichester Harbour, but in the future, more frequent counts could occur over a shorter study period (e.g., several hours a day over a period of a few weeks from a land‐based station). This would facilitate research into haul‐out patterns in relation to temporal and environmental variables (Cordes et al., 2011); aspects of harbour seal pupping (Cordes & Thompson, 2013; Reijnders et al., 2010); and the frequency and extent of human disturbance events (Andersen et al., 2012). Given the high levels of anthropogenic activity within the Solent, the latter is of particular concern. Differing patterns of environmental conditions or human use may also go some way to explaining the differential site use between Chichester and Langstone Harbours.

### 4.3 Site fidelity

Better understanding of the ecology of the Solent seals is particularly important because these animals are not all one‐off visitors; approximately 25% of harbour seals were resighted across three years, showing preliminary evidence of site fidelity by harbour seals in Chichester Harbour. Combined with the occurrence of pupping at this site, this indicates that Chichester Harbour is an area of special importance to harbour seals in the Solent. This has management implications, particularly with regard to human activities within Chichester Harbour.

In comparison, only one grey seal was resighted during our study. This may represent the more transient nature of grey seals, as well as the relatively small number of individuals in the catalogue. Although a maximum count of 20 grey seals was obtained, only eight individuals were identified. While the pelage of female grey seals is generally well marked, males can sometimes be too poorly patterned or darkly colored to allow reliable identification. Additionally, in the current study, the pelage of many seals was obscured due to clinging sediment resulting from animals hauling out on intertidal mudflats. This was particularly true for the stomach and chest regions, but less so for the back. Thus, it may be worth exploring other techniques for the collection of photographic data, such as the use of drones.

In future years, it would be beneficial to not only continue the collection of photo‐ID data in Chichester Harbour, but also expand it to include Langstone Harbour. This would allow further investigation into local site use and fidelity (Cordes & Thompson, 2015), as well as providing long‐term datasets for examination of population dynamics (Cordes & Thompson, 2014; Koivuniemi et al., 2016), reproductive patterns (Cordes & Thompson, 2013; Thompson & Wheeler, 2008), and behavior (Neumann, 1999; Wilson & Jones, 2018).

## 5 CONCLUSION

This long‐term study shows that numbers of harbour and grey seals are increasing in the Solent. This is contrary to several other populations around the UK, where harbour seals are stable or declining, but similar to increasing seal numbers in nearby southeast England and France. The south England SMU has previously been considered to have relatively few harbour seals, resulting in its omission from regular national surveys and associated reporting. However, our study suggests that this may in fact be an increasingly important area for harbour seals, based on their growing numbers, the presence of reproductive activities, and evidence of site fidelity by some individuals. Thus, it would be beneficial to continue monitoring within this SMU at a range of temporal scales and consider the resulting trends in context with those of other haul‐out sites in both the UK and Europe. Greater knowledge of the population dynamics, habitat use, behavior, and disturbance of the Solent seals will also be beneficial for managers in terms of both seal conservation and human activities.

## CONFLICT OF INTEREST

The authors declare no competing interests.

## AUTHOR CONTRIBUTIONS

**Robyne Castles:** Conceptualization (equal); Data curation (equal); Formal analysis (equal); Investigation (equal); Methodology (equal); Writing‐original draft (equal); Writing‐review & editing (equal). **Fiona Woods:** Conceptualization (equal); Data curation (equal); Formal analysis (equal); Investigation (equal); Methodology (equal); Writing‐original draft (equal); Writing‐review & editing (equal). **Peter Hughes:** Conceptualization (equal); Data curation (equal); Investigation (equal); Methodology (equal); Writing‐review & editing (equal). **John Arnott:** Conceptualization (equal); Data curation (equal); Investigation (equal); Methodology (equal); Writing‐review & editing (equal). **Louise MacCallum:** Conceptualization (equal); Data curation (equal); Investigation (equal); Methodology (equal); Writing‐review & editing (equal). **Sarah Marley:** Conceptualization (equal); Data curation (equal); Formal analysis (lead); Investigation (equal); Methodology (equal); Supervision (lead); Visualization (lead); Writing‐original draft (equal); Writing‐review & editing (equal).

### OPEN RESEARCH BADGES

This article has been awarded Open Data, Open Materials Badges. All materials and data are publicly accessible via the Open Science Framework at https://github.com/samarley86/SolentSealCounts.

## Data Availability

The data and code used in this project are freely available from: https://doi.org/10.5061/dryad.5tb2rbp4v.

## APPENDIX 1

Summary of survey effort across Chichester and Langstone Harbours. Numbers indicate the number of surveys conducted per month. Shading indicates whether surveys were conducted only in Chichester Harbour (blue), only in Langstone Harbour (red), or in both harbours (green). Note that survey methodology changed from 2015 onward (see Section 2 for details)

**Table:**

	Jan	Feb	Mar	Apr	May	Jun	Jul	Aug	Sep	Oct	Nov	Dec
1999	1	1	1	1	1	1	1	1	1	1	1	1
2000	1	1	1	1	1	1	1	1	1	1	1	1
2001	1	1	1	1	1	1	1	1	1	1	1	1
2002	1	1	1	1	1	1	1	1	1	1	1	1
2003	1	1	1	1	1	1	1	1	1	1	1	1
2004	1	1	1	1	1	1	1	1	1	1	1	1
2005	1	1	1	1	1	1	1	1	1	1	1	1
2006	1	1	1	1	1	1	1	1	1	1	1	1
2007	1		1	1	1	1	1	1	1	1	1	1
2008	1	1	1	1	1	1	1	1	1	1	1	1
2009	1	2	2	2	2	2	2	2	2	2	2	2
2010	2	2	2	2	2	2	2	2	2	2	2	2
2011	2	2	2	2	2	2	2	2	2	2	2	2
2012	2	2	2	1	1	1	1	1	1	1		1
2013	1	1	1	1	1	1	1	1	1	1	1	
2014	1	1	1	1	1	1	1	1	1	1	1	1
2015							2	2	2			
2016						2	2	2	2			
2017						2	2	2	2			
2018			1			1	1	1	1			
2019					2	4	4	4	2			

## APPENDIX 2

Full table of individual seal sightings and resightings based on photo‐ID from Chichester Harbour in 2016–2018

**Table:**

ID	2016					2017				2018						Total
	13‐Jun	27‐Jul	24‐Aug	27‐Sep	12‐Oct	20‐Jun	17‐Jul	29‐Aug	28‐Sep	26‐Mar	20‐Jun	9‐Jul	20‐Jul	21‐Aug	3‐Sep	
Harbour seals																
SOL‐1	X															1
SOL‐2	X															1
SOL‐3		X														1
SOL‐4		X	X													2
SOL‐5		X			X						X					3
SOL‐6		X										X			X	3
SOL‐7		X										X				2
SOL‐8		X														1
SOL‐9		X														1
SOL‐10			X					X							X	3
SOL‐11			X		X				X						X	4
SOL‐12			X													1
SOL‐13			X													1
SOL‐14			X													1
SOL‐15			X													1
SOL‐16			X								X				X	3
SOL‐17			X													1
SOL‐18			X						X							2
SOL‐19			X													1
SOL‐20				X												1
SOL‐21				X												1
SOL‐22				X												1
SOL‐23				X											X	2
SOL‐24				X												1
SOL‐25				X												1
SOL‐26				X												1
SOL‐27				X					X							2
SOL‐28					X				X							2
SOL‐29					X											1
SOL‐30						X										1
SOL‐31						X										1
SOL‐32							X				X					2
SOL‐33							X									1
SOL‐34							X									1
SOL‐35								X								1
SOL‐36								X								1
SOL‐37								X							X	2
SOL‐38									X						X	2
SOL‐39									X							1
SOL‐40										X						1
SOL‐41										X						1
SOL‐42										X						1
SOL‐43										X						1
SOL‐44										X						1
SOL‐45										X						1
SOL‐46										X						1
SOL‐47										X					X	2
SOL‐48											X					1
SOL‐49											X					1
SOL‐50											X					1
SOL‐51												X				1
SOL‐52												X				1
SOL‐53												X				1
SOL‐54												X				1
SOL‐55													X			1
SOL‐56													X			1
SOL‐57													X		X	2
SOL‐58														X		1
SOL‐59														X		1
SOL‐60														X		1
SOL‐61														X		1
SOL‐62															X	1
SOL‐63															X	1
SOL‐64															X	1
SOL‐65															X	1
SOL‐66															X	1
SOL‐67															X	1
SOL‐68															X	1
Grey seals																
GR‐1		X														1
GR‐2						X										1
GR‐3									X							1
GR‐4											X			X		2
GR‐5											X					1
GR‐6													X			1
GR‐7															X	1
GR‐8															X	1
